# Supplementary material for: Inverting polar domains via electrical pulsing in metallic germanium telluride
Source: Nat Commun. 2017 Apr 12;8:15033. doi: 10.1038/ncomms15033 (PMC5394341; doi:10.1038/ncomms15033)
Supplement: Supplementary Information — Supplementary Figures, Supplementary Table, Supplementary Notes and Supplementary References [file ncomms15033-s1.pdf]

Supplementary figures

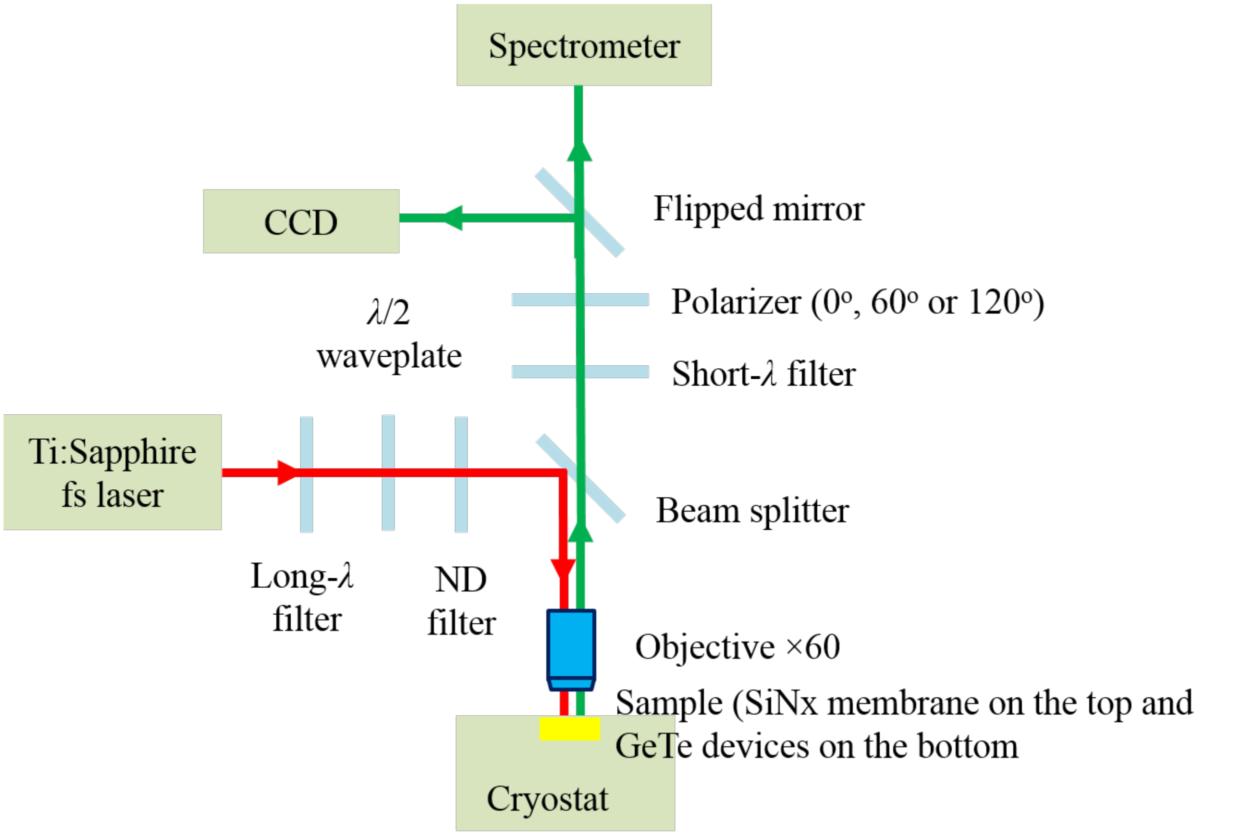

Supplementary Figure 1. Second harmonic generation polarimetry setup: Schematic of the second harmonic generation (SHG) setup used for SHG polarimetry on GeTe devices (reproduced with modification from Ref. 1).

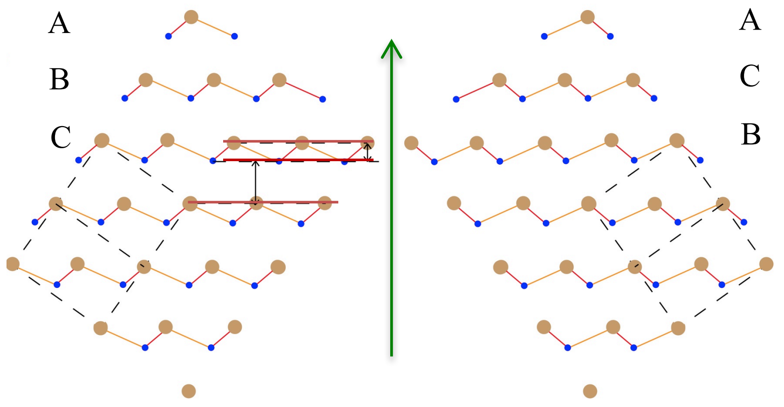

Supplementary Figure 2. Visualizing stacking domains: Schematic showing stacking twin domains (1 and 2 as referred to in the main paper), with different stacking sequences along the polarization direction. A, B and C represent different cation stacking sequences defined as in an FCC crystal. Long and short Ge (blue)-Te (yellow) bonds can be seen in this ball and stick representation. The {111} planes are represented by brown lines, and the polarization direction [111] is shown in green. These stacking domains related to each other by a rotation of 180° about the polarization direction, as can be visualized from the transformation of the dashed cell in both the domains.

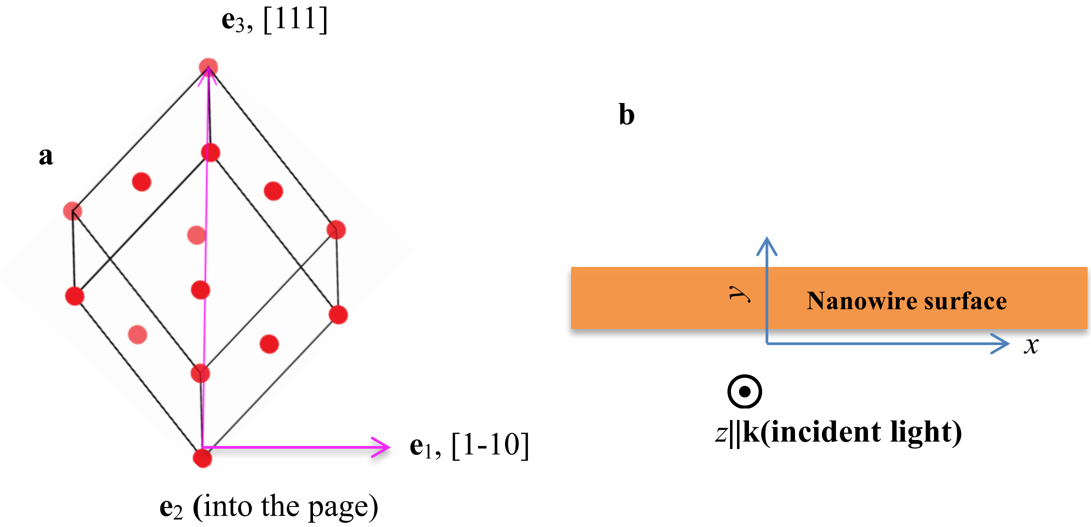

Supplementary Figure 3. Frames of reference: Description of the crystal frame of reference (a) and lab frame of reference (b) for SHG polarimetry analysis described in Supplementary Note 1. The red circles in (a) show the cationic (or anionic) positions (corners and face-centers) in a rock-salt like rhombohedral unit cell. The atoms out of page, and into the page are represented in different shades of red.

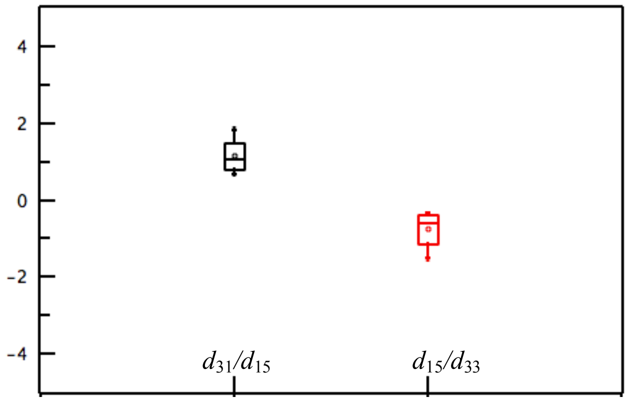

Supplementary Figure 4. **Determining $\chi^{(2)}$ tensor of GeTe** Box plot showing the values of d_{31}/d_{15} and d_{15}/d_{33} calculated from the polarimetry data on 15 different GeTe nanowires. In every box the three horizontal lines from top to the bottom of the box denote 90 percentile, the median and 10 percentile of the data respectively. The central point represents the average value, while the extreme points represent the lowest and highest estimated values of the material constants (across 15 different nanowires)

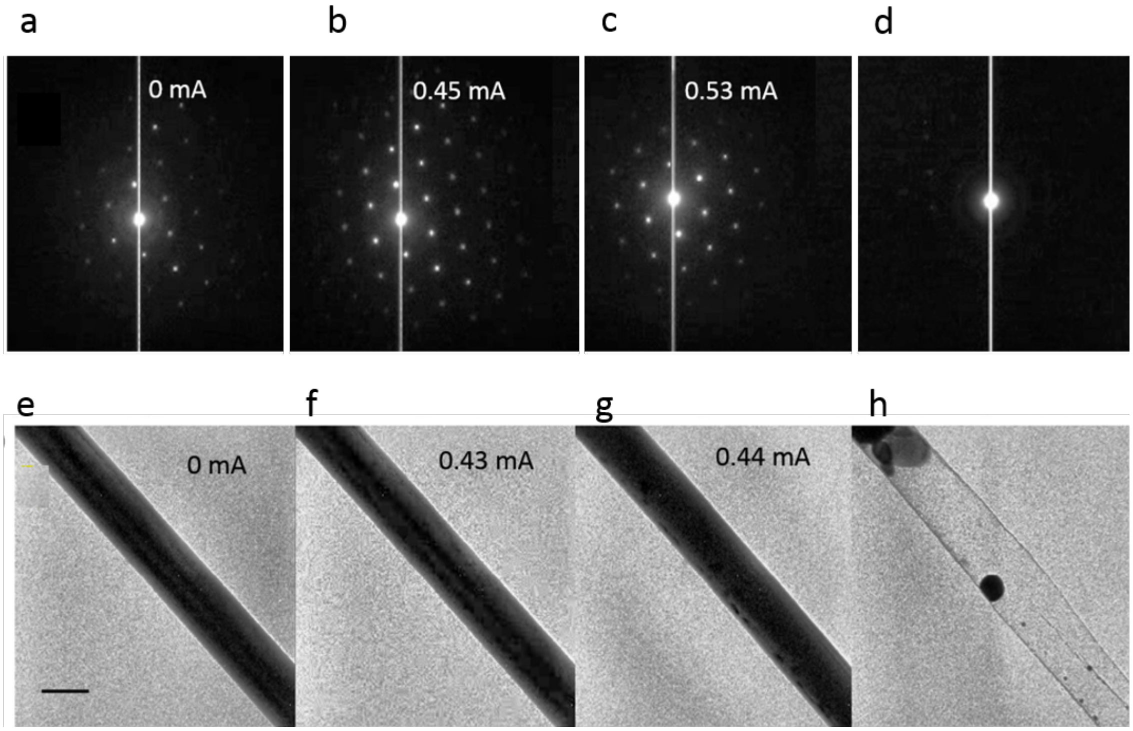

Supplementary Figure 5. More evidence that d.c. current does not couple with domain polarizations in GeTe: (a-d) Snapshots from a movie recorded in diffraction mode upon d.c. biasing until device failure. We observe no significant structural changes apart from sample-tilt effects. (e-f) Bright-field video on a thin nanowire (d ~120 nm), also showing no significant structural changes until sublimation leaving behind a shell of Al_xO_y (coated on the device via atomic layer deposition). Scale bar; 100 nm.

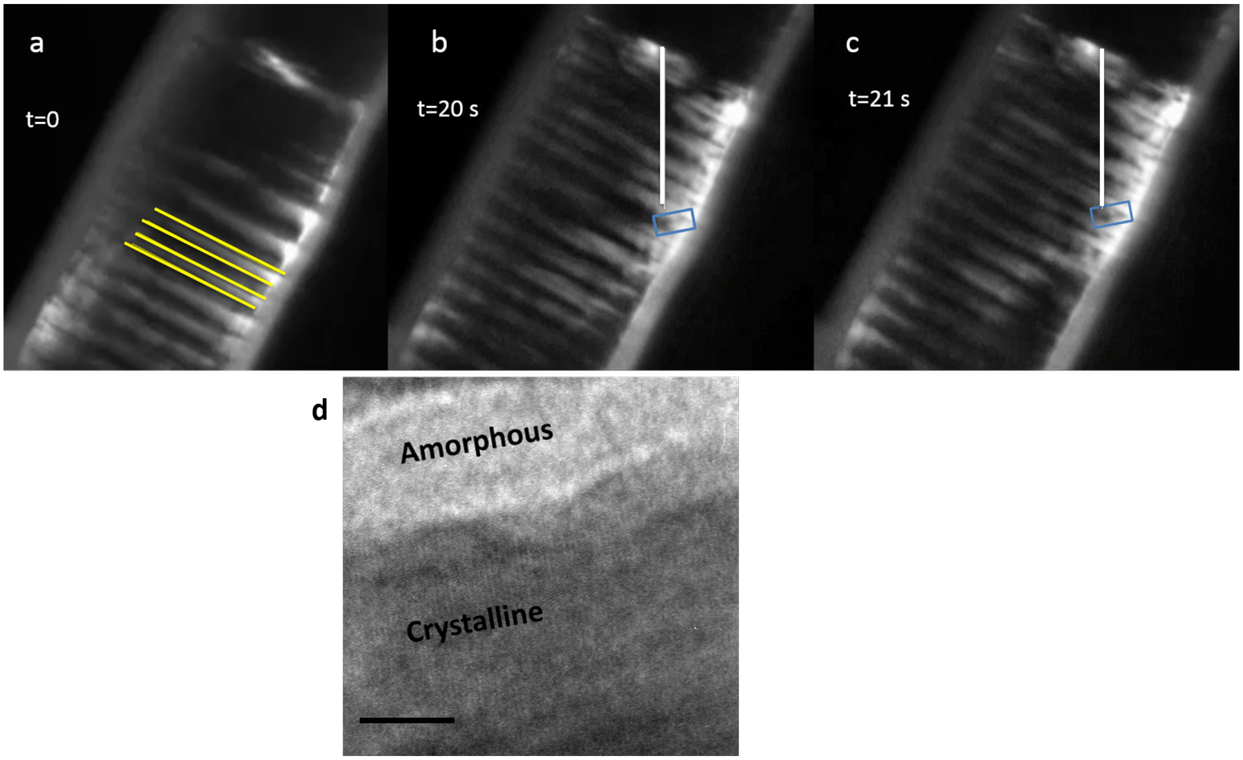

Supplementary Figure 6. **Domain boundaries, dislocations and amorphous phase in the GeTe device shown in Movie 3:** (a) Snapshots of movie 3 at t=0, clearly illustrating domain contrast. Domain boundaries are marked in yellow. (b, c) Consecutive snapshots from movie 3 at t=20 and 21 s. With respect to the reference line (white line, 175 nm) we can clearly see that the dislocation loop (line) boxed in blue moves a few nanometers in the direction of the hole-wind force. (d) HRTEM image of crystal-amorphous interface in the defect-template region after amorphization in another device. Scale bar; 5 nm.

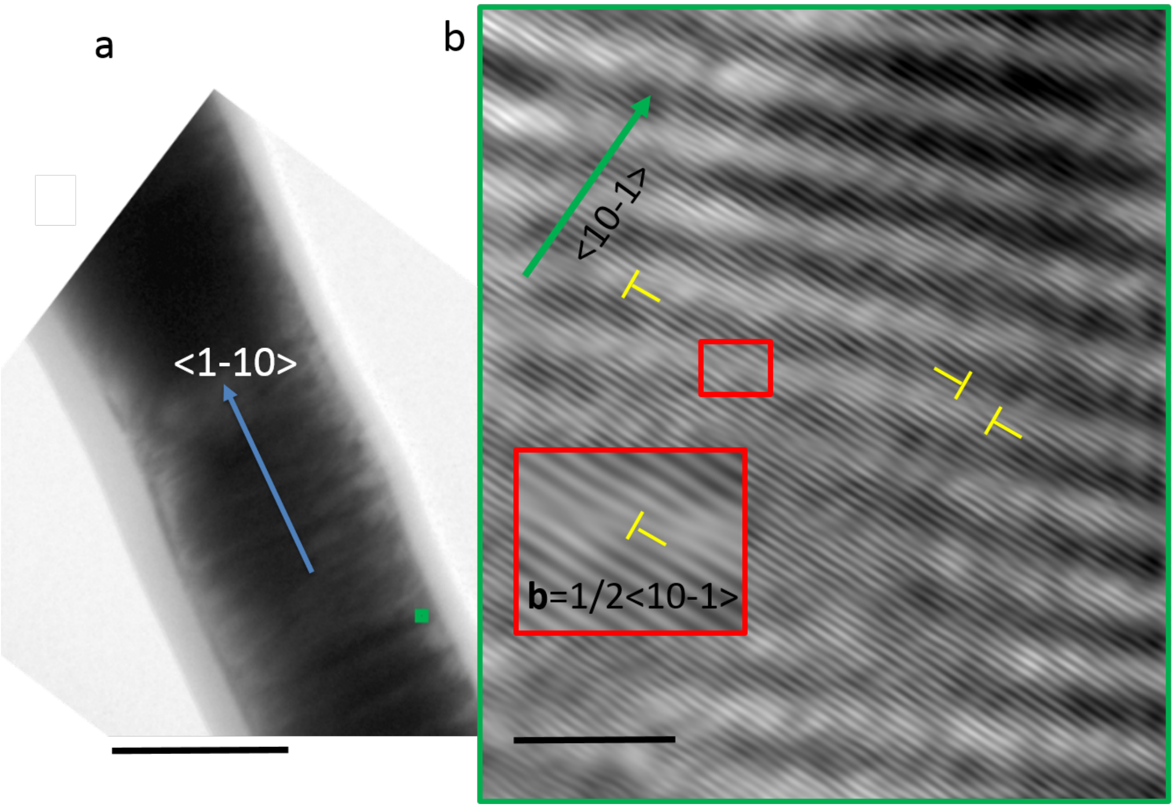

Supplementary Figure 7. HRTEM analysis of APBs and partial dislocations originating from them in programmed GeTe device: a) Bright-field image of a programmed GeTe nanowire with {1-10} domain boundaries. Image is rotated so as to correspond to the magnification rotation in the HRTEM image in (b). Growth direction of the nanowire is <1-10>. Scale bar; 200 nm. (b) Fourier processed HRTEM image near a domain boundary marked by green box in (a). We see fringes corresponding to {2 0 -2} lattice planes (*d*=2.3 A^{o}), as well as Moire-like fringes of larger periodicity. This is typical phase-contrast from stacking faults or APBs {11-1} that are not in the zone or parallel to the zone axis[2]. Some partial dislocations are identified (yellow).The partial dislocation inside the shaded red region is expanded further in the inset, and we can clearly see a partial dislocation with burgers vectors (**b**) ½<10-1> (shaded by red box). To avoid confusion the partial dislocations with **b**= ¼<10-1> are not shown. All these partial dislocations are originating from the APBs. Scale bar; 5 nm.

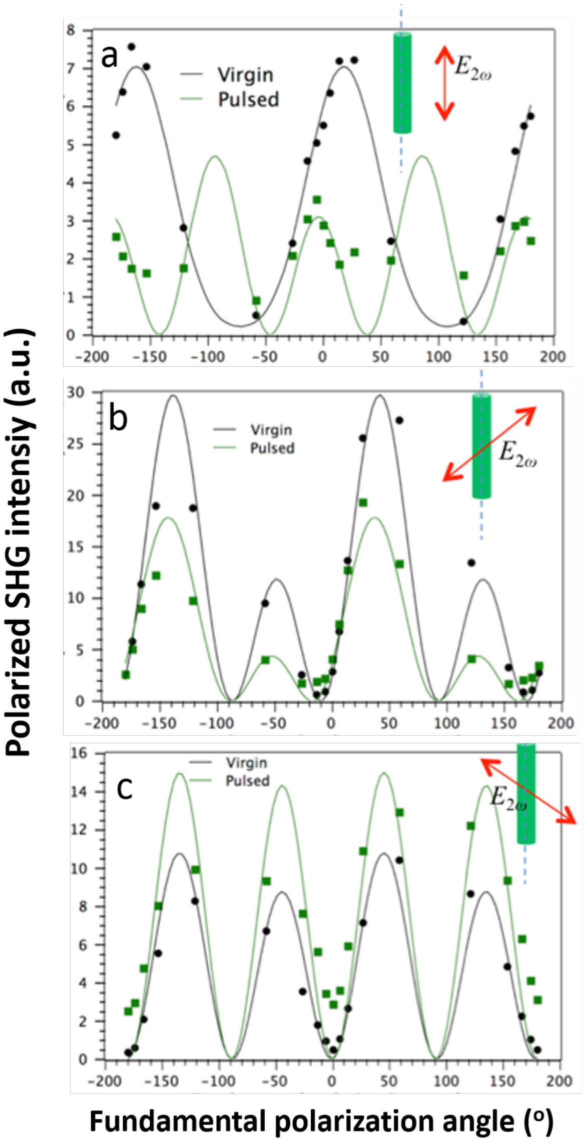

Supplementary Figure 8. SHG polarimetry on a GeTe device: SHG polarimetry data on another representative nanowire device, with diameter=650 nm and length=7 µm. (a,b,c) show the variation of intensity of SHG polarized at 0, 60 and 120° respectively with the long axis of the nanowire device with the fundamental polarization angle, and the corresponding fits (solid lines). Supplementary Table 1 shows the volume fractions of individual stacking domains and a significant inversion of the δ domains (δ_1(+) to δ_1(-) and δ_2(-) to δ_2(+) after programming, similar to the devices described in the manuscript. The formation of an APB in the δ domain near the γ, δ {001} domain boundary upon applying electrical pulses and subsequent relaxation results in inversion of polarization of the δ domain, nucleating the {110} boundaries between δ_1(-)/γ_1(-) and δ_2(+)/γ_2(+).

Supplementary Table

Volume fractions	Virgin	Programmed
$V(\beta_1):V(\gamma_1):V(\delta_1)$	1:-1.06:0.69	1:-2.05:-8.95
$V(\beta_2):V(\gamma_2):V(\delta_2)$	1:3.7:-57.33	1:5.29:19.76

Supplementary Table 1: Volume fractions of domains normalized to that of β, before and after programming. We can observe a significant inversion of the δ domain in both stacking sequences upon programming

Supplementary Notes

Supplementary Note 1. SHG polarimetry: methodology to determine domain fractions

SHG polarimetry experiment and methodology to obtain quantitative information about domain fractions was developed for GeTe, keeping in view the following problems:

1. Standard single-domain GeTe samples are lacking, and hence there is no prior characterization of the second order optical constants $\left(\chi^{(2)}\right)$ in GeTe. Single-crystalline GeTe samples, although not standard, are the best option.
2. $\chi^{(2)}$ is a third order tensor, and in the Voigt notation for any material with *R3m* symmetry written with respect to the 3-fold symmetry axis as axis '3'can be written as:

$$\chi^{(2)} = \begin{bmatrix} 0 & 0 & 0 & 0 & d_{15} & -d_{22} \\ -d_{22} & d_{22} & 0 & d_{15} & 0 & 0 \\ d_{31} & d_{31} & d_{33} & 0 & 0 & 0 \end{bmatrix} \quad (1)$$

There are 4 unknown material constants in this tensor.

3. SHG signal in single-crystalline GeTe is an interference of signals from sixteen different domains. The large number of unknown quantities (domain fractions and material constants) to be determined complicates the design of a polarimetry experiment and the analysis procedure.

Crystal and lab frames

In Voigt notation a third order tensor $\chi^{(2)}$ is written as a 3x6 matrix $[d_{ij}]$, with i=1 to 3, and j =1 to 6, and stands for:

$$\begin{cases} d_{ij} = d_{ijj}; j = 1,2,3; \\ d_{ij} = \dfrac{d_{ilm} + d_{iml}}{2}; j = 4 \Rightarrow l,m = 2,3; j = 5 \Rightarrow l,m = 3,1; j = 6 \Rightarrow l,m = 1,2 \end{cases}$$

In the coordinate system in which $\chi^{(2)}$ is written (crystal frame), $\mathbf{e}_3$ (axis-3) is the polarization direction <111>, $\mathbf{e}_1$ (axis-1) is the <110> perpendicular to the polarization direction, and $\mathbf{e}_2$ (axis-2) is a <112> direction, selected such that this coordinate system is a right-handed system (Supplementary Figure 3a).

SHG signal is collected in the lab-frame of reference, which can be defined as shown in Supplementary Fig 3b. , where the **x**-axis is the growth axis of the nanowire (NW), **z**-axis is the zone axis (**k** of the fundamental), and the **y**-axis is defined such that **x,y,z** form a right handed coordinate system.

Design of polarimetry experiment

$\alpha(+,-)$ are a pair of inversion domains with the polarization vector ($\mathbf{P}_{\alpha(+,-)}$) parallel to the zone axis (optical viewing direction). $\beta(+,-)$ are the inversion pair of domains whose polarization directions $\mathbf{P}_{\beta\ (+/-)}$ are perpendicular to the set of terminating{111}facets not parallel to the substrate. Domains $\gamma(+,-)$ and $\delta(+,-)$ have polarization directions $\mathbf{P}_{\gamma(+,-)}$, $\mathbf{P}_{\delta(+,-)}$ in the other two <111> directions respectively, not perpendicular to any of the nanowire facet (Fig. 1a). Within every FE domain, there can exist two different cationic (anionic) stacking sequences (ABCABC… or ACBACB…) along the polarization direction, resulting in two stacking domains, 1 and 2, related by 180° rotation along the polar axis (Supplementary Figure 2). Hence in totality there are sixteen domains in GeTe, $\alpha(+,-/1,2)$, $\beta(+,-/1,2)$, $\gamma(+,-/1,2)$, $\delta(+,-/1,2)$. It is important to note that there exists a three-fold rotational symmetry along the polarization axis ($\mathbf{e}_3$) in every domain.

The crystal frame of the α domains coincides with the lab frame, and hence the fundamental light is shone along $\mathbf{e}_3$ of the α domains. Owing to the three-fold symmetry along $\mathbf{e}_3$, the contribution of the α domains to the SHG signal polarized along the nanowire ($\mathbf{x_0}$), at 60° and 120° with respect to the nanowire growth axis is the same. However, in the other domains where lab frame does not coincide with the crystal frame, light is not shone along $\mathbf{e}_3$, resulting in their differential contribution to the SHG signal polarized at 0° ($\mathbf{x_0}$), 60° ($\mathbf{x_{60}}$) and 120° ($\mathbf{x_{120}}$) with respect to the nanowire according to their volume fractions. This property deconstructs individual volume fractions, and motivated us to perform the polarimetry experiment where SHG signal polarized along $\mathbf{x_0}$, $\mathbf{x_{60}}$ and $\mathbf{x_{120}}$ is collected, as a function of fundamental wave polarization angle (-180° to 180°).

Methodology:

In every domain, we transformed electric field vector of the fundamental wave from the lab frame into the crystal frame, solved for the SHG polarization vector using the equation $P(2\omega) = \chi^{(2)} E(\omega).E(\omega)$ in the crystal frame, and then transformed this vector back to lab frame. This enabled an analytical formulation of x-component of the SHG signal in the lab frame as a function of domain fractions, and material constants; which was then compared to the experimentally measured x-polarized SHG signal. In the lab frame the fundamental wave can be described in a phasor notation (with ϕ being the fundamental wave polarization angle, with the NW long axis as ϕ =0) as:

$$E = \begin{bmatrix} E_1 \\ E_2 \\ E_3 \end{bmatrix} = \begin{bmatrix} \cos(\phi) \\ -\sin(\phi) \\ 0 \end{bmatrix} E_o \qquad (2)$$

Contribution of α domains to x-polarized SHG signal: α domains comprise of four domains, α(+,-/1,2); and for these domains, the crystallographic frame is the same as the lab frame. If we assume $\chi^{(2)}$ for α(+,1) domain to be

$$\chi^{(2,\alpha(1,+))} = d_{22}\begin{bmatrix} 0 & 0 & 0 & 0 & a & -1 \\ -1 & 1 & 0 & a & 0 & 0 \\ b & b & c & 0 & 0 & 0 \end{bmatrix} \tag{3}$$

where *a,b,c* are the unknown material constants. By carrying out a rotation operation by 180° about axis $\mathbf{e}_3$, we obtain:

$$\chi^{(2,\alpha(2,+))} = d_{22}\begin{bmatrix} 0 & 0 & 0 & 0 & a & 1 \\ 1 & -1 & 0 & a & 0 & 0 \\ b & b & c & 0 & 0 & 0 \end{bmatrix} \tag{4}$$

Also for the inversion domains α(-/1,2), $\chi^{(2)}$ s are given as $\chi^{(2,\alpha(1,-))} = -\chi^{(2,\alpha(1,+))}$ and $\chi^{(2,\alpha(2,-))} = -\chi^{(2,\alpha(2,+))}$. By performing $P(2\omega) = \chi^{(2)}E(\omega).E(\omega)$ for each of these α domains, we obtain $P_x(2\omega,\alpha) = d_{22}\sin 2\phi[(V_{\alpha(1,+)} - V_{\alpha(1,-)}) - (V_{\alpha(2,+)} - V_{\alpha(2,-)})]$, where V_i is the volume of domain '*i*'. From here on for convenience we refer to $(V_{\alpha(1,+)} - V_{\alpha(1,-)})$ as α_1, and $(V_{\alpha(2,+)} - V_{\alpha(2,-)})$ as α_2, and likewise with $\beta_{1,2}$, $\gamma_{1,2}$ and $\delta_{1,2}$. So the contribution of α domains to x-polarized SHG signal is**:**

$$P_{x0}(2\omega,\alpha) = d_{22}\sin 2\phi[\alpha_1 - \alpha_2] \tag{5}$$

Contribution of β, γ and δ domains to x-polarized (0°) SHG signal: For β domains, the crystal axis can be obtained from the lab axis with a rotation vector, R_β, given as

$$R_\beta = \begin{pmatrix} 1 & 0 & 0 \\ 0 & 1/3 & -2\sqrt{2}/3 \\ 0 & 2\sqrt{2}/3 & 1/3 \end{pmatrix} \tag{6}$$

So $P(2\omega,\beta)$ can be calculated as $R_\beta^{-1}(\chi^{(2,\beta(1,2/+,-))}R_\beta E)$ by transformation of fundamental wave 'E' to the crystal frame first, evaluation of SHG signal in the crystal frame, and transforming it back to the lab frame. This gives

$$P_{x0}(2\omega,\beta) = d_{22}\sin 2\phi\left[1/3(\beta_1 - \beta_2) + 2\sqrt{2}/3\, a(\beta_1 + \beta_2)\right] \tag{7}$$

Similarly $P(2\omega,\gamma)$ and $P(2\omega,\delta)$ can be evaluated where

$$R_\gamma = \begin{pmatrix} 1/2 & -\sqrt{3}/2 & 0 \\ 1/2\sqrt{3} & 1/6 & -2\sqrt{2}/3 \\ \sqrt{2}/\sqrt{3} & \sqrt{2}/3 & 1/3 \end{pmatrix} \tag{8}$$

and

$$R_\delta = \begin{pmatrix} -1/2 & -\sqrt{3}/2 & 0 \\ 1/2\sqrt{3} & -1/6 & -2\sqrt{2}/3 \\ \sqrt{2}/\sqrt{3} & -\sqrt{2}/3 & 1/3 \end{pmatrix} \tag{9}$$

$$P_{x0}(2\omega,\gamma) = \begin{cases} d_{22}\sin 2(\phi)\left[(0.24a+0.31b-0.31c)(\gamma_1+\gamma_2)-0.22(\gamma_1-\gamma_2)\right] \\ +d_{22}\sin^2(\phi)\left[(-0.41a+0.64b+0.18c)(\gamma_1+\gamma_2)-0.06(\gamma_1-\gamma_2)\right] \\ +d_{22}\cos^2(\phi)\left[(0.41a+0.27b+0.54c)(\gamma_1+\gamma_2)-0.19(\gamma_1-\gamma_2)\right] \end{cases} \tag{10}$$

and

$$P_{x0}(2\omega,\delta) = \begin{cases} d_{22}\sin 2(\phi)\left[(-0.24a-0.31b+0.31c)(\delta_1+\delta_2)+0.22(\delta_1-\delta_2)\right] \\ +d_{22}\sin^2(\phi)\left[(-0.41a+0.64b+0.18c)(\delta_1+\delta_2)-0.06(\delta_1-\delta_2)\right] \\ +d_{22}\cos^2(\phi)\left[(0.41a+0.27b+0.54c)(\delta_1+\delta_2)+0.19(\delta_1-\delta_2)\right] \end{cases} \tag{11}$$

Finally, $P_{x0}(2\omega) = P_{x0}(2\omega,\alpha) + P_{x0}(2\omega,\beta) + P_{x0}(2\omega,\gamma) + P_{x0}(2\omega,\delta)$ (12)

and

$$I_{x0}(2\omega) = [P_{x0}(2\omega)]^2 = \left[d_{22} \left\{ \begin{array}{l} \cos^2\phi \begin{bmatrix} -0.19[((\gamma_1-\gamma_2)-(\delta_1-\delta_2)]+ \\ (0.41a+0.27b+0.54c)[((\gamma_1+\gamma_2)+(\delta_1+\delta_2)] \end{bmatrix} \\ \sin^2\phi \begin{bmatrix} -0.06[((\gamma_1-\gamma_2)+(\delta_1-\delta_2)]+ \\ (-0.41a+0.64b+0.18c)[((\gamma_1+\gamma_2)+(\delta_1+\delta_2)] \end{bmatrix} \\ \sin 2\phi \begin{bmatrix} (\alpha_1-\alpha_2)+0.33(\beta_1-\beta_2)-0.22[(\gamma_1-\gamma_2)-(\delta_1-\delta_2)] \\ +0.94a(\beta_1+\beta_2)+ \\ (0.24a+0.31b-0.31c)[(\gamma_1+\gamma_2)-(\delta_1+\delta_2)] \end{bmatrix} \end{array} \right\} \right]^2$$

(13)

Note the simple contributions to the signal from *α* and *β* domains whose polarization vectors are perpendicular to $\mathbf{x_0}$, and the more complicated contributions from γ and δ whose polarizations are not perpendicular.

Contributions of individual domains to 60° polarized (with respect to the nanowire long axis) SHG signal: To evaluate the SHG signal polarized at 60° with respect to the nanowire long axis, we chose our lab axis, such that **x, y** axis are rotated by 60° in plane ($\mathbf{x_{60}}$, $\mathbf{y_{60}}$) with respect to the nanowire long axis and '**z**' remains as in the previous case. In this lab-frame of reference, we note that there is no change to the signal from *α* domains because of the three-fold symmetry in this domain about the **z**-axis. However, the contribution of *γ*, *δ* and *β* domains to SHG polarized along $\mathbf{x_{60}}$ is the same as the contribution of *β*, *γ* and *δ* domains respectively to the SHG polarized along $\mathbf{x_0}$, evaluated in the previous case with $\mathbf{x_0}$, $\mathbf{y_0}$, and **z** forming the basis vectors of lab-frame. Now signal from *γ* domain shows pure sin 2*(ϕ*-60*)* dependence, whereas the signal from *δ* and *β* domains shows dependence on all the second order sine and cosine functions. The following expressions can be written from these permutations:

$$P_{x60}(2\omega,\alpha) = d_{22}\sin 2(\phi-60)[\alpha_1-\alpha_2] \tag{14}$$

$$P_{x60}(2\omega,\gamma) = d_{22}\sin 2(\phi-60)\left[\tfrac{1}{3}(\gamma_1-\gamma_2) + \tfrac{2\sqrt{2}}{3}a(\gamma_1+\gamma_2) \right]$$

(15)

$$P_{x60}(2\omega,\delta)=\begin{cases} d_{22}\sin 2(\phi-60)\left[(0.24a+0.31b-0.31c)(\delta_1+\delta_2)-0.22(\delta_1-\delta_2)\right] \\ +d_{22}\sin^2(\phi-60)\left[(-0.41a+0.64b+0.18c)(\delta_1+\delta_2)-0.06(\delta_1-\delta_2)\right] \\ +d_{22}\cos^2(\phi-60)\left[(0.41a+0.27b+0.54c)(\delta_1+\delta_2)-0.19(\delta_1-\delta_2)\right] \end{cases}$$

(16)

$$P_{x60}(2\omega,\beta)=\begin{cases} d_{22}\sin 2(\phi-60)\left[(-0.24a-0.31b+0.31c)(\beta_1+\beta_2)+0.22(\beta_1-\beta_2)\right] \\ +d_{22}\sin^2(\phi-60)\left[(-0.41a+0.64b+0.18c)(\beta_1+\beta_2)-0.06(\beta_1-\beta_2)\right] \\ +d_{22}\cos^2(\phi-60)\left[(0.41a+0.27b+0.54c)(\beta_1+\beta_2)+0.19(\beta_1-\beta_2)\right] \end{cases}$$

(17)

$$P_{x60}(2\omega)=P_{x60}(2\omega,\alpha)+P_{x60}(2\omega,\beta)+P_{x60}(2\omega,\gamma)+P_{x60}(2\omega,\delta)$$

(18)

$$I_{x60}(2\omega)=[P_{x60}(2\omega)]^2=\left[d_{22}\left\{\begin{matrix} \cos^2(\phi-60)\begin{bmatrix}-0.19[((\delta_1-\delta_2)-(\beta_1-\beta_2)]+ \\ (0.41a+0.27b+0.54c)[(\delta_1+\delta_2)+(\beta_1+\beta_2)]\end{bmatrix} \\ \sin^2(\phi-60)\begin{bmatrix}-0.06[((\delta_1-\delta_2)+(\beta_1-\beta_2)]+ \\ (-0.41a+0.64b+0.18c)[(\delta_1+\delta_2)+(\beta_1+\beta_2)]\end{bmatrix} \\ \sin 2(\phi-60)\begin{bmatrix}(\alpha_1-\alpha_2)+0.33(\gamma_1-\gamma_2)-0.22[(\delta_1-\delta_2)-(\beta_1-\beta_2)] \\ +0.94a(\gamma_1+\gamma_2)+ \\ (0.24a+0.31b-0.31c)[(\delta_1+\delta_2)-(\beta_1+\beta_2)]\end{bmatrix} \end{matrix}\right\}\right]^2$$

(19)

Contributions of individual domains to 120° polarized (w.r.t. NW long axis) SHG signal: Finally, for 120° SHG polarization, the lab-frame is defined by the basis vectors $\mathbf{x_{120}}$, $\mathbf{y_{120}}$, and $\mathbf{z}$. α and δ domains will give a simple sin2(ϕ-120) contribution, whereas β and γ do not. The contributions of δ, β and γ's to the SHG signal polarized along $\mathbf{x_{120}}$, are one cyclic permutation of the previous case.

$$P_{x120}(2\omega,\alpha)=d_{22}\sin 2(\phi-120)[\alpha_1-\alpha_2] \quad (20)$$

$$P_{x120}(2\omega,\delta) = d_{22}\sin 2(\phi-120)\left[{}^{1}\!/_{3}(\delta_1-\delta_2) + {}^{2\sqrt{2}}\!/_{3}\, a(\delta_1+\delta_2) \right]$$

(21)

$$P_{x120}(2\omega,\beta) = \begin{cases} d_{22}\sin 2(\phi-120)\left[(0.24a+0.31b-0.31c)(\beta_1+\beta_2)-0.22(\beta_1-\beta_2)\right] \\ +d_{22}\sin^2(\phi-120)\left[(-0.41a+0.64b+0.18c)(\beta_1+\beta_2)-0.06(\beta_1-\beta_2)\right] \\ +d_{22}\cos^2(\phi-120)\left[(0.41a+0.27b+0.54c)(\beta_1+\beta_2)-0.19(\beta_1-\beta_2)\right] \end{cases}$$

(22)

$$P_{x120}(2\omega,\gamma) = \begin{cases} d_{22}\sin 2(\phi-120)\left[(-0.24a-0.31b+0.31c)(\gamma_1+\gamma_2)+0.22(\gamma_1-\gamma_2)\right] \\ +d_{22}\sin^2(\phi-120)\left[(-0.41a+0.64b+0.18c)(\gamma_1+\gamma_2)-0.06(\gamma_1-\gamma_2)\right] \\ +d_{22}\cos^2(\phi-120)\left[(0.41a+0.27b+0.54c)(\gamma_1+\gamma_2)+0.19(\gamma_1-\gamma_2)\right] \end{cases}$$

(23)

Finally, $P_{x120}(2\omega) = P_{x120}(2\omega,\alpha) + P_{x120}(2\omega,\beta) + P_{x120}(2\omega,\gamma) + P_{x120}(2\omega,\delta)$,

(24)

$$I_{x120}(2\omega) = [P_{x120}(2\omega)]^2 = \left[d_{22} \left\{ \begin{array}{l} \cos^2(\phi-120)\left[\begin{array}{l} -0.19[((\beta_1-\beta_2)-(\gamma_1-\gamma_2)]+ \\ (0.41a+0.27b+0.54c)[(\beta_1+\beta_2)+(\gamma_1+\gamma_2)] \end{array}\right] \\ \sin^2(\phi-120)\left[\begin{array}{l} -0.06[((\beta_1-\beta_2)+(\gamma_1-\gamma_2)]+ \\ (-0.41a+0.64b+0.18c)[(\beta_1+\beta_2)+(\gamma_1+\gamma_2)] \end{array}\right] \\ \sin 2(\phi-120)\left[\begin{array}{l} (\alpha_1-\alpha_2)+0.33(\delta_1-\delta_2)-0.22[(\beta_1-\beta_2)-(\gamma_1-\gamma_2)] \\ +0.94a(\delta_1+\delta_2)+ \\ (0.24a+0.31b-0.31c)[(\beta_1+\beta_2)-(\gamma_1+\gamma_2)] \end{array}\right] \end{array} \right\} \right]^2$$

(25)

Experimentally, by varying the fundamental wave polarization (ϕ), we measure I_{x0}, $I_{x\text{-}60}$ and $I_{x\text{-}120}$. From Supplementary eqns. 13, 19 and 25, we note that $I_{x\theta}$, where θ can be 0, 60 or 120° is of the form:

$$I_{x\theta}(2\omega) = [A_\theta \cos^2(\phi-\theta) + B_\theta \sin^2(\phi-\theta) + C_\theta \sin 2(\phi-\theta)]^2 \quad (26)$$

A_θ, B_θ and C_θ are functions of material constants and domain fractions. It must be noted that owing to the large number of unknowns and limited data, we can estimate only the following quantities: d_{31}/d_{15}, d_{15}/d_{33}, $V_{d,i}/V_{\alpha,i}$ where d can be β, γ or δ, and 'i' can be 1 or 2, with the understanding that $V_{d,i}=V_{d,i}(+)-V_{d,i}(-)$, where d is any ferroelastic domain. Specifically, this analysis does not give the ratios of stacking domains $V_{d,1}/V_{d,2}$, as determining them needs the knowledge of another material constant d_{31}/d_{22}, which also cannot be estimated.

Algorithm to obtain domain volume fractions and non-linear optical material constants:
The algorithm to obtain material constants and domain fractions is as follows:

1. Fit the acquired data to Supplementary eq. 26, and obtain all the nine coefficients A_θ, B_θ and C_θ with θ = 0, 60 or 120°.
2. By comparing numerical values of all the nine extracted coefficients with the coefficients of Supplementary eqns. 13, 19, and 25, we obtain nine bilinear equations in nine variables: d_{31}, d_{15}, d_{33}, $V(\beta_1)/V(\alpha_1)$, $V(\beta_2)/V(\alpha_2)$, $V(\gamma_1)/V(\alpha_1)$, $V(\gamma_2)/V(\alpha_2)$, *and* $V(\delta_1)/V(\alpha_1)$, $V(\delta_2)/V(\alpha_2)$.
3. Solve for self-consistent values of all the nine variables using MATLAB.

On 15 different nanowire devices of large diameters (>500 nm to avoid anisotropy in in-coupling and out-coupling of light); we estimated the median value of the material constants d_{31}/d_{15}, and d_{15}/d_{33} to be 1.05 and -0.53 respectively (Supplementary Figure 4).

Supplementary Note. 2: Identification of various types of defects in GeTe

Dislocation motion and domain contrast can be identified in dark-field images (and also bright-field), and in particular from the movies, as long as the domains boundaries are in the zone of observation. When the {1-10} boundaries are formed, they can be identified as the boundary between darker contrast regions (arising out of less intense diffraction spot of the double spot) and the brighter contrast regions (arising out of more intense diffraction spot in the **g**=<02-2> double spot). Supplementary Figure 6 shows snapshots at different times in the Movie 3. At t=0 (Suppelmentary Figure 6a) we mark these domain boundaries in a straightforward manner, and the domain contrast is very clear.

Dislocations can be identified as one dimensional loops or lines (as in Fig. 3j in the manuscript near the defect template after recrystallization). We point out how to identify dislocation motion in the dark-field movies, by following a partial in Movie 3 which moves a few nanometers in the direction of hole-wind force in consecutive frames at t=20 sec and t=21 sec (Supplementary Figure 6 b,c). The 175 nm reference line which starts at the reference point (notch), clearly elucidates the motion of this dislocation (although it is also obvious in the movie). Since there are a large number of partials following similar dynamics, in the video it appears as a net contrast motion in the direction of hole-wind force, but careful observation allows us to point out to individual dislocation contrast and follow it.

The amorphous phase in the dark-field images appear as a really bright line, due to large dynamic scattering of the defect template surrounding it. Infact, diffraction near the template (Fig.

4e in the manuscript) shows very intense Bragg reflections (more intense than the central spot itself). So, a thumb rule to identify amorphous phase in in situ dark-field videos is to look for the brightest region corresponding to the moment of resistance rise in the programming curve. We confirmed the amorphous phase and crystal-amorphous interface using HRTEM on another nanowire device (Supplementary Figure 6d).

APBs (stacking faults which separates the crystal into two parts differing by an phase of π in structure factor) on the other hand, cannot be identified via diffraction contrast TEM[2]. Phase-contrast TEM is the best way to identify them, and Supplementary Fig 6b shows HRTEM images of APB near a {110} domain boundary. APB's ({11-1} plane) which are not in the zone (<111>), can be identified by their Moire-fringe like patterns as can be seen in Supplementary Figure 7b (the non-lattice fringes of larger periodicity[2]). By following the lattice-fringes in this region, we could also identify several partial dislocations with burgers vectors <101>/2 and <101>/4 that originate from the APBs.

Supplementary References:

1. Ren, M.-L., Liu, W., Aspetti, C. O., Sun, L. & Agarwal, R. Enhanced second-harmonic generation from metal-integrated semiconductor nanowires via highly confined whispering gallery modes. *Nat. Commun.* **5,** 5432 (2014).

2. Fultz, B. & Howe, J. M. *Transmission electron microscopy and diffractometry of materials*. Springer (2008).
